# Fractal Analysis and Artificial Intelligence for Radiographic Detection of Periodontal Bone Loss: A Systematic Review

**DOI:** 10.3390/diagnostics16050782

**Published:** 2026-03-05

**Authors:** Zülal Deniz Güner, Merter Güçlü, Fatma Karacaoğlu, Nilsun Bağış, Kaan Orhan

**Affiliations:** 1 Department of Periodontology, Faculty of Dentistry, Ankara University, Ankara 06560, Turkey; fboke@ankara.edu.tr (F.K.); nbagis@ankara.edu.tr (N.B.); 2 Department of Periodontology, Faculty of Dentistry, Bozok University, Yozgat 66100, Turkey; merter.guclu@bozok.edu.tr; 3 Department of Dento Maxillofacial Radiology, Faculty of Dentistry, Ankara University, Ankara 06560, Turkey; call53@yahoo.com; 4 Medical Design Application and Research Center (MEDITAM), Ankara University, Ankara 06560, Turkey; 5 Department of Oral Radiology, School and Hospital of Stomatology, Cheeloo College of Medicine, Shandong University, Jinan 250012, China

**Keywords:** artificial intelligence, fractal analysis, periodontitis, radiographic bone loss, systematic review

## Abstract

**Background/Objectives**: Accurate diagnosis and staging of periodontitis rely on clinical measurements and radiographic assessment of alveolar bone loss. **Methods**: Studies published between 1 January 2020 and 31 October 2025 were searched in the Web of Science and PubMed databases in accordance with the PRISMA guidelines. Original research articles that evaluated periodontal pathology on radiographic images using fractal analysis and/or artificial intelligence approaches, with clearly defined methodologies, were included. Due to methodological heterogeneity, a quantitative meta-analysis was not performed, and the findings were summarized using a narrative synthesis approach. **Results**: Of 346 records, 80 studies (9 fractal, 71 AI) met the inclusion criteria. Fractal analysis studies predominantly calculated the fractal dimension on panoramic or periapical radiographs using the box-counting method. In artificial intelligence studies, the task types mainly comprised classification, segmentation, detection, and hybrid approaches (multi-stage models or models combining multiple tasks). Panoramic and intraoral radiographs were the predominant imaging modalities. Performance metrics were reported across wide ranges (sensitivity 0.23–1.00; accuracy 0.506–1.00; specificity 0.41–0.99; F1 score 0.15–0.99; AUC 0.75–0.99), and in some studies, these metrics were only partially reported. **Conclusions**: Fractal analysis and artificial intelligence approaches offer objective and reproducible assessment of periodontal bone loss; however, methodological and reporting heterogeneity limit comparability and generalizability. Standardization of ROI definitions, datasets, study designs, and performance reporting is needed to improve clinical applicability. Future research should also explore hybrid models that combine the quantitative microstructural insights of fractal analysis with the automated detection capabilities of artificial intelligence to enhance diagnostic precision.

## 1. Introduction

Periodontitis is a chronic inflammatory disease characterized by progressive destruction of the periodontal tissues, resulting from the complex interaction between bacterial infection and the host immune response [1]. Accurate diagnosis and appropriate classification of the disease are critical for effective treatment planning and determination of long-term prognosis. In current clinical practice, the diagnosis of periodontitis relies on clinical and radiological parameters, including periodontal pocket depth measurements, the presence of bleeding on probing, and radiographic assessment of alveolar bone levels [2]. However, these evaluations may lead to inconsistencies due to observer-dependent variability, reliance on clinical experience, and the time-consuming nature of the measurements [3,4].

Accurate and reliable determination of alveolar bone loss plays a central role in both the diagnosis of periodontitis and the monitoring of disease progression [5]. Although radiographic examination is widely used in clinical practice, its sensitivity is limited, particularly in detecting subtle early-stage bone changes [6]. This limitation has highlighted the need for complementary analytical methods capable of a more sensitive and quantitative assessment of microstructural changes in periodontal bone tissue.

In this context, fractal analysis (FA), which enables mathematical characterization of the complex architecture of bone, has emerged as a quantitative approach of growing interest in periodontal research. Fractal analysis expresses the structural complexity of bone tissue through the fractal dimension (FD) [7,8]. The relative independence of FA from projection geometry and radiodensity variations, its noninvasive nature, and its ability to provide objective data on trabecular bone structure constitute important advantages [9,10]. In the literature, fractal analysis has been applied across various clinical domains, including the evaluation of periodontal diseases, monitoring of healing after root canal treatment, assessment of bone regeneration following orthognathic surgery, and analysis of dental caries [11,12,13,14].

In recent years, rapid advances in digital technologies have led to the increasingly widespread use of artificial intelligence (AI)–based approaches in healthcare. Artificial intelligence refers to systems capable of mimicking certain cognitive processes characteristic of human intelligence by processing information through algorithms and large datasets [15]. Owing to developments in image processing, AI applications have become powerful tools supporting diagnostic processes in dentistry [16,17]. Current literature reports the use of AI across a wide range of dental applications, including periodontal assessment and detection of bone loss, as well as caries detection, restoration analysis, cephalometric evaluation, orthognathic surgery planning, and implant planning [18,19].

By virtue of their ability to automatically learn complex patterns from large datasets, AI systems have the potential to reduce observer-dependent variability, improve time efficiency, and enhance diagnostic accuracy in the diagnosis and classification of periodontitis [20]. In this respect, fractal analysis and AI approaches may be regarded as complementary methods. While fractal analysis generates quantitative structural features from radiographic images, AI models can leverage these features and/or raw image data to achieve high performance in classification, segmentation, or regression-based analyses. Therefore, a combined and systematic evaluation of the literature on fractal analysis and AI applications in periodontal diseases is warranted.

The aim of this systematic review is to synthesize the existing studies on fractal analysis and AI-based approaches for the diagnosis of periodontal diseases and to provide a comprehensive overview of the methodological approaches employed, the reported diagnostic performance metrics, and the existing gaps in the literature.

## 2. Materials and Methods

### 2.1. Literature Search

This systematic review was conducted in accordance with the Preferred Reporting Items for Systematic Reviews and Meta-Analyses (PRISMA) guidelines (Table S2), and the study protocol was prospectively registered in the International Prospective Register of Systematic Reviews (ID: 1278324). The literature search was conducted for studies published between 1 January 2020 and 31 October 2025 in the Web of Science and PubMed databases. This time period was chosen to cover the period when deep learning-based approaches became widespread in periodontal imaging and to increase methodological homogeneity. The search strategy was created by combining relevant keywords using Boolean operators (AND, OR, NOT). The detailed search strategy used is summarized in Table 1. In the PubMed search, both MeSH terms and title/abstract keywords were used together. This approach aims to increase the sensitivity of the search by including new publications that have not yet been fully indexed with MeSH terms. 

### 2.2. Eligibility Criteria

The research question of this systematic review was to evaluate the diagnostic use of fractal analysis and/or artificial intelligence-based approaches applied to radiographic images in individuals with periodontal pathology. Specifically, it was investigated whether these approaches provide meaningful diagnostic performance when compared with clinical or radiographic reference standards.

According to the PICO framework, the population comprised individuals with periodontal pathology. With respect to the interventions, studies employing fractal analysis methods or artificial intelligence algorithms, including machine learning and deep learning, for the assessment of periodontal pathology were included. For the comparison, studies incorporating conventional clinical and radiographic assessment methods (e.g., periodontal pocket depth measurements, bleeding on probing, radiographic indices, or manual analyses) were considered; additionally, studies without a direct comparison group but reporting diagnostic performance metrics (such as accuracy, sensitivity, specificity, or AUC) were also included.

The inclusion criteria encompassed original research articles published in English that used fractal analysis and/or artificial intelligence methods for the radiological evaluation of periodontal diseases, provided clear methodological descriptions, and included statistical analyses. Case reports, case series, narrative reviews, systematic reviews, meta-analyses, conference abstracts, and editorial articles were excluded, as were animal or cell-based in vitro studies, duplicate publications, methodologically inappropriate studies, and articles for which the full text was unavailable. Data related to the predefined outcome measures were systematically extracted from the included studies.

### 2.3. Data Extraction

Data extraction from the selected studies was performed independently by three investigators (Z.D.G., F.K., and M.G.) using a predefined standardized data extraction form. For each study, basic descriptive information was first recorded, including the study title, year of publication, study design, and sample size. With respect to fractal analysis, the imaging modality used, region of interest (ROI) selection, and reported fractal analysis outcomes were extracted. For artificial intelligence applications, the task type (classification, segmentation, and detection), characteristics and size of the dataset, and the reported diagnostic performance metrics (such as accuracy, sensitivity, specificity, precision, recall, and reproducibility) were systematically collected. Any disagreements among the investigators during the data extraction process were resolved through discussion; when consensus could not be reached, the opinion of a fourth investigator (N.B.) was sought to make the final decision.

### 2.4. Synthesis of Results

Due to substantial methodological heterogeneity among the included studies, quantitative synthesis (meta-analysis) was not feasible, and a narrative synthesis was therefore conducted. For diagnostic accuracy outcomes, effect measures reported in the original studies included sensitivity, specificity, and the area under the receiver operating characteristic curve (AUC). For fractal analysis studies, fractal dimension (FD) values were extracted as reported. No data conversion or imputation was performed. Studies with missing or non-comparable summary statistics were included only in the qualitative synthesis. Study findings were presented in tabular form to facilitate comparison of outcomes across the included studies.

### 2.5. Risk of Bias Assessment

The methodological quality and risk of bias of the studies were assessed by two independent reviewers using the updated QUADAS-2 (Quality Assessment of Diagnostic Accuracy Studies-2) tool for diagnostic accuracy studies. The assessment process covered the following four key areas: patient selection, index test, reference standard, and workflow and timing. A ‘low’, ‘high’, or ‘unclear’ risk of bias score was assigned for each domain. Disagreements between assessors were resolved through discussion; where consensus could not be reached, a third researcher’s opinion was sought. Risk of bias results are presented in tables and supported by graphical representations.

## 3. Results

### 3.1. Study Selection

**Figure 1:**
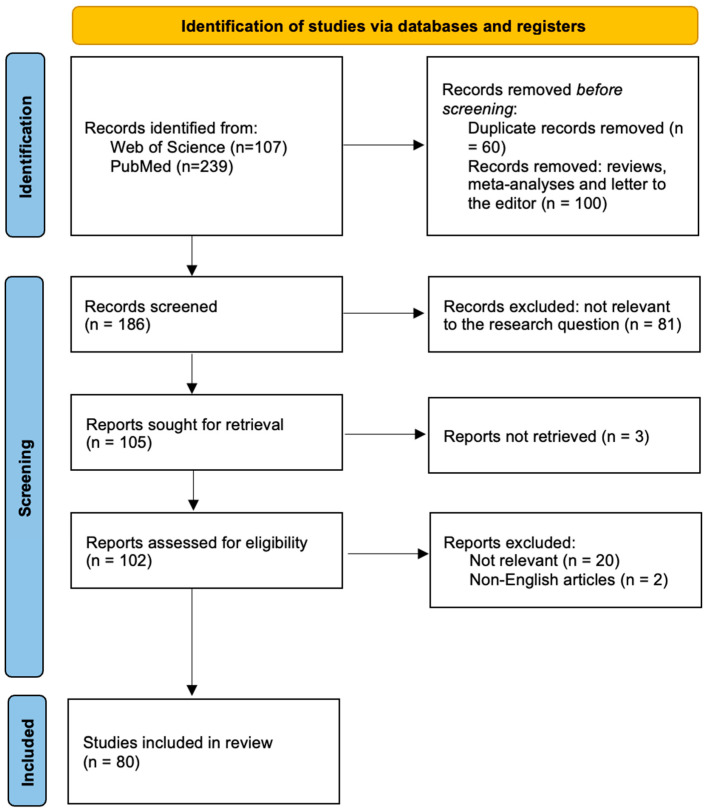
PRISMA 2020 flow diagram of study selection.

Following the literature search, a total of 346 records were identified from the electronic databases (Web of Science, *n* = 107; PubMed, *n* = 239). Prior to title and abstract screening, duplicate records (*n* = 60) and publications in the form of reviews, meta-analyses, and letters to the editor (*n* = 100) were excluded. The remaining 186 records were screened at the title and abstract level; after excluding studies not relevant to the research question, 105 articles were selected for full-text assessment. Four studies for which the full text was unavailable were excluded. Following eligibility assessment, two studies were excluded because they were not published in English, and 19 studies were excluded because they were not related to the research topic. Ultimately, a total of 80 studies that met the predefined inclusion criteria were included in the systematic review (Figure 1).

### 3.2. Risk of Bias Results

**Figure 2:**
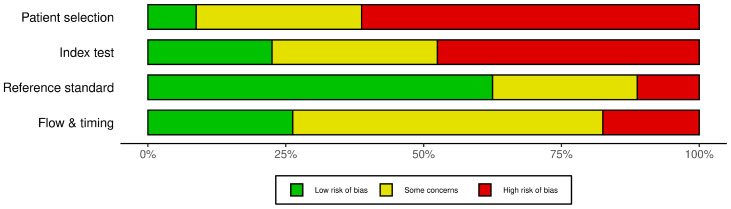
Risk of bias assessment for the included studies using QUADAS-2 domains.

The risk of bias, patient selection, index test, reference standard, and flow and timing were assessed using the QUADAS-2 tool (Figure 2 and Figure S1). The quality assessment of the included studies using the QUADAS-2 tool revealed significant methodological differences; according to the analysis results, the weakest area of the studies was “Patient Selection,” with more than half of the studies in this category carrying a high risk of bias. In contrast, the “Reference Standard” domain emerged as the lowest risk and most reliable domain, while high-risk ratios were evident in the “Index Test” domain, and uncertainties (some concerns) were more prominent in the “Flow and Timing” domain. Overall, most of the included studies exhibit a high risk of bias or some concerns in at least one area, while only a limited number of studies show low risk in all four areas [21,22]. Consequently, although the studies are generally successful in applying reference standards, the generalizability of the findings may be limited due to the high risk of bias in patient selection.

### 3.3. Study Focus and Sample

Within the scope of this systematic review, a total of 80 studies published between 2020 and 2025 were evaluated. Of these studies, 9 involved fractal analysis, while 71 focused on artificial intelligence applications. Among the studies employing fractal analysis, five compared fractal dimension (FD) values between patients with periodontitis at different stages and periodontally healthy individuals [21,23,24,25,26]. One study compared FD values between the gingivitis and periodontitis groups [27]. In the remaining three studies, fractal dimension was compared between individuals with and without gingival recession undergoing orthodontic treatment [28], across different stages of periodontitis in patients with diabetes [29], and between periodontally healthy individuals and those with periodontitis in the perimenopausal period [30]. In these studies, sample sizes ranged from 39 to 200. The main characteristics of the studies involving fractal analysis, including sample sizes, imaging modalities, ROI definitions, and reported fractal dimension values, are summarized in Table 2.

Among the 71 artificial intelligence-based studies, 51 evaluated alveolar bone loss. Six studies focused on the classification of periodontal disease [31,32,33,34,35,36], and seven examined various alveolar bone defects, including intrabony defect angles [37], vertical and horizontal bone defects, and furcation involvement [38,39,40,41,42,43]. Three studies assessed peri-implant bone loss and morphology [44,45,46], while the remaining four reported measurements of clinical attachment loss (CAL) [22], the periodontal ligament (PDL) [47], the cementoenamel junction (CEJ) [48], and alveolar crest height [49]. Sample sizes in these studies ranged from 6 to 8000.

### 3.4. Technique Employed

In the studies involving fractal analysis, three were conducted using periapical radiographs [24,26,29], while six were based on panoramic radiographs [21,23,25,27,28,30]. All fractal analysis studies used the box-counting method to calculate the fractal dimension.

Of the 71 artificial intelligence-based studies included in the systematic review, 36 applied hybrid approaches, 17 used classifications, 9 employed detection, and 5 performed segmentation. In addition, three studies reported quantitative measurement/regression-based approaches, and one study reported vision–language-based classification. Hybrid models comprised multi-stage architectures that combined classification, segmentation, and/or detection steps, or architectures that generated multiple outputs simultaneously. Moreover, a limited number of studies employed approaches that did not directly involve classification or detection, such as quantitative measurement, regression-based prediction, or vision–language-based methods; this explains why the total number of studies does not correspond exactly to the basic task categories (Table S1). Overall, these studies utilized a wide range of artificial intelligence methods for periodontal and alveolar bone assessment. The applied approaches were predominantly based on deep learning architectures, with convolutional neural network (CNN)-based models (ResNet, VGG, DenseNet, Inception, MobileNet, and EfficientNet), U-Net and its variants, the YOLO family, R-CNN-based detection models, and Transformer/ViT-based approaches being widely reported in the literature. In addition, some studies reported the use of GAN-based architectures, two-stage CNN structures, and hybrid CNN–machine learning (CNN–ML) models.

Most of these studies were conducted using panoramic and intraoral radiographs. Panoramic images constituted the most frequently used imaging modality, followed by periapical and bitewing radiographs. Additionally, a limited number of studies reported the use of cone-beam computed tomography (CBCT) data. Details regarding the algorithms, imaging modalities, and task types used in the artificial intelligence approaches are summarized in Table 3.

### 3.5. Reference Standard

In the included artificial intelligence studies, the reference standard was predominantly defined based on manual segmentation, annotation, or classification performed by experienced dentists, periodontists, oral and maxillofacial (OMF) radiologists, or clinicians. In the majority of the studies, the gold standard was established through independent assessments by two or more experts or by consensus agreement.

A total of nine studies conducted model-to-model comparisons; in one study, an image-based model (OPG), a clinical data-based model (EHR), and a multimodal fusion model (OPG + EHR) were compared. In addition, one study compared the performance of an artificial intelligence model with the assessments of undergraduate and final-year dental students. In six studies, the comparator or reference standard was not explicitly specified. Details regarding the reference standards and comparison approaches used are presented in Table 3.

### 3.6. Measurement Method

In the included studies, periodontal bone loss was generally calculated as relative bone loss percentage (RBL%), derived from linear measurements based on the distance between the cementoenamel junction (CEJ) and the alveolar crest or apex. In some studies, these measurements were further used to classify periodontal disease. In other studies, the presence and characteristics of alveolar bone defects (including the presence and classification of furcation defects, vertical and horizontal bone defects, and intrabony defect angles), clinical attachment loss (CAL), the periodontal ligament (PDL), the cementoenamel junction (CEJ), and alveolar crest height were evaluated.

In the fractal analysis studies, substantial heterogeneity was observed in the selection of regions of interest (ROI). ROI sizes ranged from 15 × 15 pixels to 60 × 60 pixels, and in some studies, the ROI size was not explicitly reported [24,26,29]. ROIs were most commonly positioned in the mandibular posterior regions. The most frequently selected anatomical sites included the interradicular region between the mandibular second premolar and first molar; the mesial, distal, and apical regions of mandibular first molars; interdental areas; and supracortical bone regions. Additionally, some studies evaluated the apical regions of mandibular incisors, canines, and premolars; the mesial and distal surfaces of right and left mandibular molars; condylar and gonial regions; and bilateral areas between the first molar and second premolar as ROIs. In one study, ROI dimensions were reported to be region-specific, defined as 60 × 60 pixels and 20 × 100 pixels [30].

### 3.7. Reported Fractal Dimension Values

The included studies reported that fractal dimension (FD) values varied according to periodontal health status. In periodontally healthy individuals and regions, FD values were reported to range approximately between 1.21 and 1.66 and were generally higher than those observed in the presence of periodontal disease. In contrast, individuals with periodontal disease typically exhibited lower FD values, although the reported ranges varied widely, approximately between 1.02 and 1.86.

Some studies indicated a gradual decrease in FD values with increasing disease severity [23,24,26,29,30]. However, one study reported no statistically significant difference in FD values between the gingivitis and periodontitis groups, with reported values of 1.195 ± 0.121 and 1.196 ± 0.141, respectively [27].

### 3.8. Metrics for Evaluating AI-Based Diagnostic Performance

The diagnostic performance metrics of artificial intelligence-based models showed wide variability across studies. The reported sensitivity values ranged from 0.23 to 1.00, accuracy from 0.506 to 1.00, specificity from 0.41 to 0.99, precision from 0.11 to 0.99, recall from 0.26 to 1.00, and F1 scores from 0.15 to 0.99. In studies reporting the area under the curve (AUC), values ranged between 0.75 and 0.99. However, only a subset of these performance metrics was reported in some of the included studies, and in seven studies, none of these metrics were reported. Overall, artificial intelligence-based models showed moderate to high diagnostic performance for the detection, quantification, and staging of periodontal bone loss across different radiographic modalities. The direction of effect consistently favored AI-assisted or automated approaches over unaided human assessment, with particularly strong performance in mild-to-moderate disease, and results often comparable to those of experienced clinicians. However, diagnostic accuracy varied by disease severity and task complexity. Detailed information on the performance metrics and reported outcomes of the artificial intelligence models is provided in Table 3.

**Table 2:** Characteristics of fractal-analysis studies included in the review.

Authors	Sample Size	Imaging Method	Periodontal Disease	ROI Size	ROI Area	Measurement Method	Clinical Examination	Fractal Dimension (Mean ± SD)	
**Dosdoğru et al., 2025 [21]**	39	Panoramic	20 Periodontally Healthy—19 Stage III/IV Periodontitis	30 × 30 pixels	Interradicular area: left mandibular 2nd premolar–1st molar	Box-counting	Yes	Periodontally Healthy = 1257.40 ± 70.64 Stage III/IV Periodontitis = 1227.79 ± 69.79	
**Yarkaç et al., 2023 [23]**	200	Panoramic	50 periodontally healthy, 50 Stage 1 periodontitis, 50 Stage 2 periodontitis, 50 Stage 3 periodontitis	50 × 50 pixels	Interradicular area: mandibular 2nd premolar–1st molar	Box-counting	Yes (PI, GI, PPD, CAL)	Periodontally healthy = 1.44 ± 0.06 Stage 1 periodontitis = 1.36 ± 0.08 Stage 2 periodontitis = 1.35 ± 0.07 Stage 3 periodontitis = 1.28 ± 0.15	
**Mishra et al., 2023 [24]**	75	Periapical	15 periodontally healthy, 15 Stage 1 periodontitis, 15 Stage 2 periodontitis, 15 Stage 3 periodontitis,15 Stage 4 periodontitis	NA	Interdental area	Box-counting	Yes (PD, CAL)	Periodontally healthy = 1.21 ± 0.07 Stage 1 periodontitis = 1.21 ± 0.06, Stage 2 periodontitis = 1.19 ± 0.05 Stage 3 periodontitis = 1.11 ± 0.05 Stage 4 periodontitis = 1.02 ± 0.11	
**Korkmaz et al., 2023 [25]**	61	Panoramic	33 periodontally healthy, 28 stage III/IV grade C periodontitis	15 × 15 pixels	Mesial or distal to the mandibular canine, Mesial or distal to the first mandibular molar, Supracortical bone above the antegonial notch	Box-counting	Yes (PD, CAL)	Mandibular molar	PH =1.51 ± 0.07 P. = 1.37 ± 0.13
								Mandibular canine	PH =1.50 ± 0.07 P. = 1.40 ± 0.14;
								Antegonial notch	PH =1.50 ± 0.08 P. = 1.39 ± 0.14
**Soltani et. al., 2021 [26]**	80	Periapical	36 periodontally healthy, 8 mild, 13 moderate, and 13 severe periodontitis	NA	Mandibular first molars: mesial, distal and apical mandibular first molars	Box-counting	Yes (CAL)	Apical	PH = 1.66 (1.50–1.69) Mild P. = 1.62 (1.54–1.67) Moderate P. = 1.52 (1.38–1.62) Severe P. = 1.32 (1.24–1.55)
								Mesial	PH = 1.63 (1.59–1.66) Mild P. = 1.60 (1.56–1.64) Moderate P. = 1.55 (1.43–1.64) Severe P. = 1.54 (1.42–1.58)
								Distal	PH = 1.60 (1.55–1.63) Mild P. = 1.57 (1.51–1.61) Moderate P. = 1.58 (1.53–1.59) Severe P. = 1.42 (1.36–1.56)
**Eser et. al., 2024 [27]**	128	Panoramic	64 gingivitis, 64 periodontitis	23 × 51 pixels	left or right mandibular molar: mesial, distal	Box-counting	No	Gingivitis = 1.195 ± 0.121 Periodontitis = 1.196 ± 0.141	
**Küçükoğlu Çolak et al., 2025 [28]**	60	Panoramic	Gingival Recession Periodontally Healthy (Control)	70 × 35 pixels	Apical region: left (G1)-right (G2) mandibular incisor-canine left (G3)-right (G4) premolar	Box-counting	Yes (PI, GI)	T0	G1 = 1.3680 ± 0.059/G1c = 1.3812 ± 0.052 G2 = 1.3546 ± 0.11/G2c = 1.3959 ± 0.062 G3 = 1.3792 ± 0.104/G3c = 1.3919 ± 0.051 G4 = 1.3809 ± 0.098/G4c = 1.3819 ± 0.054
**Kayaalti-Yüksek et al., 2024 [29]**	125	Periapical	DM patients with mild-moderate periodontitis, DM patients with advanced periodontitis, SH individuals with mild-moderate periodontitis, SH individuals with advanced periodontitis, SH individuals with gingivitis (control group).	NA	Mandibular first molars: mesial, distal	Box-counting	Yes (PPD, CAL, BOP, Stages of periodontitis)	Distal	DM-MMP = 1.78 ± 0.08 DM-AP = 1.78 ± 0.08 SH-MMP = 1.81 ± 0.07 SH-AP = 1.80 ± 0.06 SH-G = 1.85 ± 0.06
								Mesial	DM-MMP = 1.77 ± 0.07 DM-AP = 1.80 ± 0.09 SH-MMP = 1.78 ± 0.015 SH-AP = 1.78 ± 0.11 SH-G = 1.86 ± 0.05
**Eninanç and Bostancı, 2025 [30]**	60	Panoramic	Perimenopausal and periodontally healthy (PERI-H); perimenopausal with periodontitis (PERI-P); postmenopausal and periodontally healthy (POST-H); postmenopausal with periodontitis (POST-P)	60 × 60 pixels, 60 × 60 pixels, 20 × 100 pixels	Right and left condylar regions, Right and left gonial regions, The region between the first molar and second premolar teeth bilaterally	Box-counting	Yes+ Mandibular Cortical Index (MCI), Mental Index (MI), Panoramic Mandibular Index (PMI)	Right condylar regions	PERI-H = 1.622 ± 0.042 PERI-P = 1.569 ± 0.061 POST-H = 1.542 ± 0.059 POST-P = 1.545 ± 0.099
								Left condylar regions	PERI-H = 1.620 ± 0.055 PERI-P = 1.575 ± 0.086 POST-H = 1.567 ± 0.064 POST-P = 1.575 ± 0.083
								Right gonial regions	PERI-H = 1.596 ± 0.088 PERI-P = 1.548 ± 0.125 POST-H = 1.527 ± 0.119 POST-P = 1.533 ± 0.113
								Left gonial regions	PERI-H = 1.568 ± 0.071 PERI-P = 1.565 ± 0.099 POST-H = 1.557 ± 0.083 POST-P = 1.524 ± 0.144
								Right interdental regions	PERI-H = 1.334 ± 0.041 PERI-P = 1.296 ± 0.104 POST-H = 1.279 ± 0.048 POST-P = 1.269 ± 0.041
								Left interdental regions	PERI-H = 1.568 ± 0.071 PERI-P = 1.565 ± 0.099 POST-H = 1.284 ± 0.065 POST-P = 1.258 ± 0.032

**Table 3:** Characteristics of AI studies included in the review.

Authors	Al Algorithm	Radiographic Imaging	Periodontal Parameter	Sensitivity	Accuracy	Inference/Main Finding
**Cerda Mardini et al., 2024 [50]**	Deep CNN	Panoramic	PBL	0.23	NA	Model achieved acceptable performance for mild–moderate PBL but failed for severe PBL.
**Yu et al., 2024 [51]**	SegFormer	Panoramic	PBL	0.7347	0.8674	SegFormer-PCA achieved high correlation with clinicians for RBL measurement.
**Kabir et al., 2022 [52]**	U-Net with ResNet-34	Periapical	Staging %RBL	Stage I 0.99 Stage II 0.66 Stage III 0.88	NA	AI accurately assigned tooth numbers and staged periodontitis on intraoral radiographs and reliably arranged full-mouth series images.
**Kearney et al., 2022 [22]**	GAN (CNN) Deep Lab V3 + DETR	Bitewing-Periapical	CAL	NA	NA	GAN-based inpainting significantly reduced CAL prediction error compared with non-inpainted methods and achieved ≈1 mm accuracy versus clinical measurements.
**Li et al., 2024 [53]**	Unet & TransUNet & TA-Net & CE-Net & DS-TransUNet	Panoramic	Periodontitis Segmentation (Upper/Lower Alveolar Bone Loss Regions)	0.9736 (periodontitis segmentation, TA-Net, Test3)	0.9833 (classification, ResNet, mixed dataset)	Multi-center panoramic dataset enabled high-performance benchmarking for periodontitis segmentation and classification tasks.
**Zhao et al., 2022 [54]**	Modified BCDU-Net & Dual-loss autoencoder & DNN	Panoramic	Extent of bone loss, Maximum bone loss, Age-adjusted bone loss	0.93 (Chapter III); 0.84 (Chapter VI); 0.77 (Chapter IX)	0.88 (Chapter III); 0.82 (Chapter VI); 0.72 (Chapter IX)	Multimodal deep learning using panoramic radiographs and EHR data predicted systemic disease chapters III, VI, and IX with the highest performance.
**Li et al., 2025 [31]**	HC-Net, HC-Net+	Panoramic	Periodontal disease classification	0.956	0.924	HC-Net+ accurately detected stage II–IV periodontitis on panoramic radiographs and outperformed clinicians.
**Jiang et al., 2022 [55]**	DL-UNET & YOLOv4	Panoramic	Staging %PBL + Vertical Resorption + Furcation	0.77	0.77	Two-stage deep learning model accurately staged radiographic periodontal bone loss and outperformed general dentists.
**Jundaeng et al., 2025 [56]**	YOLOv8 (CNN)	Panoramic	Alveolar bone loss	1.00	0.98	AI accurately segmented CEJ and alveolar bone levels and enabled individualized periodontal prognosis on panoramic radiographs.
**Camlet et al., 2025 [57]**	ChatGPT	Panoramic	PBL (Remaining Bone Height)	85.8–93.1% (tooth presence detection; model-dependent)	NA	ChatGPT models (4.5, o1, o3 ve o4-mini-high) overestimated remaining bone height and showed insufficient accuracy for periodontal classification despite acceptable tooth count agreement.
**Kurt-Bayrakdar et al., 2025 [38]**	nnU-Net v2	CBCT	Types of periodontal bone defects (total alveolar bone loss, supra-bony defects, infra-bony defects, perio–endo lesions, buccal defects, and furcation defects)	0.60 (total alveolar bone loss segmentation)	0.99 (segmentation models); 0.80 (healthy)/0.76 (unhealthy) classification	AI automatically segmented teeth and multiple periodontal bone defect types and classified periodontal health status on CBCT images.
**Butnaru et al., 2025 [58]**	CNNs	Panoramic	PBL (Alveolar + Structural Indicators)	NA	NA	AI performance in detecting alveolar bone loss and attachment loss was comparable to that of senior specialists on panoramic radiographs.
**Liu et al., 2025 [59]**	Mask R-CNN & U-Net	Panoramic	PBL	NA	0.9073	AI achieved 90.73% accuracy for staging periodontitis on panoramic radiographs.
**AlGhaihab et al., 2025 [60]**	Denti.AI (DL) ResNeT, FPN	Bitewing- Periapical	RBL	NA	NA	Deep learning assistance did not significantly improve dentists’ detection of radiographic bone loss.
**Li et al., 2021 [61]**	Deetal-Perio, R-CNN	Panoramic	Alveolar bone loss (ABL), Periodontitis prediction	NA	0.892 (Suzhou dataset); 0.819 (Zhongshan dataset)	Interpretable AI accurately predicted periodontitis severity using alveolar bone loss features on panoramic radiographs.
**Resul et al., 2025 [32]**	APD-FFNet	Panoramic	Periodontitis (binary classification: healthy vs. periodontitis)	NA	0.94	The APD-FFNet model holds considerable promise for clinical application, particularly in automated periodontitis diagnosis.
**Mema et al., 2025 [62]**	Diagnocat	Panoramic	PBL (With Associated Dental Findings)	0.96	0.96	Diagnocat showed high sensitivity but moderate specificity for detecting periodontal bone loss on panoramic radiographs.
**Shetty et al., 2024 [39]**	ResNet50 & ResNet101 & ResNet101V2	CBCT	Furcation Involvement	NA	0.91	ResNet101V2 accurately detected furcation involvement on axial CBCT images.
**Chang et al., 2022 [63]**	DL-multitasking InceptionV3 model	Periapical	RBL Classification	0.86 ± 0.03	0.87 ± 0.01	Deep learning accurately classified mild versus severe radiographic bone loss on periapical radiographs.
**Zadrożny et al., 2022 [64]**	Diagnocat AI (CNN-based system)	Panoramic	PBL	0.801	NA	AI showed acceptable reliability for detecting periodontal bone loss on panoramic radiographs.
**Alotaibi et al., 2022 [65]**	DL-CNN-based model VGG-16	Periapical	Alveolar Bone Loss	0.73	0.7304	CNN detected alveolar bone loss and classified severity levels on periapical radiographs with moderate accuracy.
**Jundaeng et al., 2025 [66]**	YOLOv8 (CNN)	Panoramic	PBL	1.00	0.98	AI accurately segmented CEJ and alveolar bone levels and enabled automated staging of periodontitis on panoramic radiographs.
**Uzun Saylan et al., 2023 [67]**	YOLOv5x	Panoramic	PBL (Local + Total)	0.75	NA	AI achieved higher performance for regional alveolar bone loss detection than for total alveolar bone loss on panoramic radiographs.
**Ibraheem et al., 2025 [68]**	Second Opinion	Periapical	Marginal Bone Loss	91.1% (marginal bone loss)	NA	AI software showed high diagnostic accuracy for detecting marginal bone loss on periapical radiographs.
**Vollmer et al., 2023 [69]**	Mask R-CNN & ResNet-50-FPN	Panoramic	Alveolar Bone Region (Keypoint-Based Localization Only)	NA	NA	AI detected maxillary posterior molars and quantified radiographic bone loss using keypoint-based measurements on panoramic radiographs.
**Ryu et al., 2023 [70]**	Faster R-CNN	Panoramic	PBL	0.84	NA	DL model reliably detected periodontal bone loss on panoramic radiographs using regional tooth grouping.
**Putra et al., 2025 [71]**	YOLOv8	Panoramic	PBL	0.9464	0.9027	YOLOv8 accurately detected periodontal bone loss on panoramic radiographs.
**Kong et al., 2023 [72]**	Two-stage PDCNN architecture	Panoramic	RBL (Staging: Healthy/Mild/Moderate/Severe)	NA	0.762	Two-stage PDCNN achieved 0.762 accuracy for multi-class radiographic bone loss classification on panoramic radiographs.
**AlGhaihab et al., 2025 [73]**	DL-ResNet CNN	Bitewing- Periapical	RBL	0.76 (periapical); 0.65 (bitewing)	0.81 (periapical); 0.76 (bitewing)	Denti.AI demonstrated clinically acceptable performance for detecting radiographic alveolar bone loss on intraoral radiographs.
**Chen et al., 2023 [74]**	CNN: YOLOv5 including VGG-16 and U-Net	Bitewing- Periapical	Remaining interproximal bone level detection and RBL detection	NA	0.9261 (bone level detection); 0.970 (radiographic bone loss detection)	DL ensemble model accurately detected tooth position, bone level, and radiographic bone loss on periapical radiographs.
**Su et al., 2024 [47]**	Res-Net	CBCT	PDL segmentation	NA	96.4% (molars); 100% (incisors, canines, premolars, wisdom teeth, implants)	Mask R-CNN enabled automatic PDL segmentation on CBCT with mIoU 0.667 and mDSC 0.799.
**Dujic et al., 2023 [75]**	Vit Base & VitLarge & BeiTBase & BEiT Large & DeiTbase	Periapical	PBL	0.898–0.913 (overall, depending on model)	0.834–0.852 (overall)	Vision transformer networks achieved good performance for automated detection of periodontal bone loss on periapical radiographs.
**Vilkomir et al., 2024 [40]**	ResNet-18	Periapical	Classification of mandibular molar furcation involvement	0.95	0.96	ResNet-18 accurately classified mandibular molar furcation involvement on periapical radiographs.
**Yavuz et al., 2024 [33]**	YOLOv8-cls (Ultralytics)	Bitewing- Periapical	Classification of periodontal status	0.8243 (bitewing); 0.7500 (periapical)	0.7703 (bitewing); 0.7500 (periapical)	DL algorithm classified periapical and bitewing radiographs as periodontally healthy or diseased with ≥75% accuracy.
**Piroonsan et al., 2025 [41]**	InceptionV3, InceptionResNetV2, ResNet50V2, MobileNetV3Large, EfficientNetV2B1, and VGG19	Bitewing- Periapical	Three-Wall Intrabony Defects	0.67 (VGG19, dataset B3)	0.78 (VGG19, dataset B3)	CNN models classified three-wall intrabony defects on intraoral radiographs with acceptable AUC values.
**Ezhov et al., 2021 [76]**	CNN	CBCT	PBL	0.9239	NA	AI-assisted CBCT diagnosis significantly improved dentists’ sensitivity and specificity compared with unaided evaluation.
**Schulze et al., 2024 [42]**	CNN-Diagnocat	CBCT	Periodontal lesions (Vertical bone defect/mixed periodontal bone loss/furcation lesion)	NA	NA	CNN showed lower performance than human observers in detecting periodontal lesions on CBCT datasets.
**Guler Ayyildiz et al., 2024 [77]**	ResNet50 & DenseNet121 & InceptionV3	Panoramic	PBL Classification	0.833	0.907	DenseNet121-based model accurately classified periodontal bone loss stages on panoramic radiographs.
**Bahadır et al., 2025 [78]**	DentisToday	Panoramic	PBL	NA	0.936 (periodontal loss identification by AI)	AI showed higher accuracy than undergraduate students in identifying periodontal loss on panoramic radiographs.
**Widyaningrum et al., 2022 [79]**	Multi-Label U-Net & Mask R-CNN	Panoramic	RBL (Staging 1–4)	0.88 (Mask R-CNN, average recall)	0.95 (Mask R-CNN detection accuracy)	Multi-Label U-Net achieved superior segmentation performance compared with Mask R-CNN on panoramic radiographs.
**Dai et al., 2024 [80]**	AlexNet, VGG16, ResNet18 with multiple classification algorithms (RF, SVM, NB, LR, KNN)	Periapical	PBL	0.915 (PER-AlexNet model)	0.872 (PER-AlexNet model)	CNN combined with classification algorithms enabled accurate staging of periodontitis using periapical radiographs.
**Chen et al., 2024 [81]**	U-Net + Mask-RCNN (deep CNN pipeline)	Periapical	PBL measurement & staging	0.97 (cut-off 0.15); 0.952 (cut-off 0.33)	0.728 (overall staging accuracy)	CNN-based system quantified periodontal bone loss and staged periodontitis on periapical radiographs.
**Erturk et al., 2025 [82]**	YOLOv8	Bitewing	PBL	NA	0.83446 (test, average)	YOLOv8 accurately classified periodontal bone loss stages on bite-wing radiographs.
**Zhang et al., 2023 [44]**	CNN (ResNet-50)	Panoramic	Peri-Implant Marginal Bone Loss (With/Without Bone Loss)	NA	0.870 (hybrid model)	DL model predicted dental implant failure using periapical and panoramic radiographs.
**Mao et al., 2023 [83]**	CNN (GoogLeNet; AlexNet; VGG19; Inception v3)	Panoramic	RBL (Site, Type, Severity)	0.956 (GoogLeNet)	0.9497 (GoogLeNet)	CNN accurately detected furcation involvement on periapical radiographs.
**Li et al., 2025 [84]**	YOLOv8	Panoramic	RBL	NA	0.725 (overall stage classification)	YOLOv8-assisted model staged radiographic bone loss on panoramic radiographs.
**Liu et al., 2023 [85]**	CNN (PAR-CNN model based on AlexNet)	Panoramic	PBL, Periodontitis Stage	0.795	0.790	CNN model detected periodontitis on panoramic radiographs with diagnostic performance comparable to that of periodontal experts.
**Xue et al., 2024 [86]**	YOLOv8 & Mask R-CNN & TransUNet	Panoramic	PBL	NA	0.8945 (overall stage classification)	DL method accurately diagnosed periodontal bone loss and periodontitis stage on panoramic radiographs.
**Shon et al., 2022 [48]**	U-Net & YOLOv5	Panoramic	CEJ Detection (No Periodontal Bone Loss Measurement)	NA	0.928	Integrated U-Net and YOLOv5 framework accurately classified periodontitis stages on panoramic radiographs.
**Almarghlani et al., 2025 [87]**	ML, Second Opinion^®^	Bitewing- Periapical	Alveolar Bone Loss	NA	NA	AI measurements showed stronger correlation with radiographic measurements than dental practitioners’ estimates.
**Hoss et al., 2023 [88]**	ResNet-18, MobileNet V2, ConvNeXT/large	Periapical	PBL	0.907 (highest: ConvNeXT/base)	0.848 (highest: ConvNeXT/base)	CNNs detected periodontal bone loss on periapical radiographs with similar diagnostic performance.
**Kurt-Bayrakdar et al., 2024 [89]**	CNN U-Net	Panoramic	PBL (Total + Vertical + Horizontal + Furcation)	1.000 (total bone loss)	0.994 (total bone loss)	AI accurately detected periodontal bone loss patterns and furcation defects on panoramic radiographs.
**Li et al., 2024 [34]**	YOLOv4 + CNN (AlexNet)	Bitewing	Periodontal Diseases	NA	0.921 (periodontitis recognition, AlexNet)	AI model detected periodontal disease on bitewing radiographs.
**Ertaş et al., 2023 [90]**	kNN, ANN, SVM, RF, NB, LR (clinical data); DenseNet121, EfficientNetB0, InceptionV3, ResNet50, VGG16 (image DL); hybrid CNN+ML	Panoramic	RBL (%), % bone loss/age, CAL, Vertical/horizontal bone loss	NA	0.972 (tree algorithm, clinical data—staging)	Machine learning models enabled automated staging and grading of periodontitis using clinical data and panoramic radiographs.
**Orhan et al., 2023 [91]**	Diagnocat CNN Software	Panoramic	PBL	0.818	NA	AI software detected periodontal bone loss on panoramic radiographs.
**Amasya et al., 2024 [92]**	Mask R-CNN (ResNet-101 backbone) + Cascade R-CNN	Panoramic	PBL	0.999 (binary bone loss detection, overall)	0.980 (binary bone loss detection, overall)	AI software accurately detected periodontal bone loss on panoramic radiographs.
**Do et al., 2025 [93]**	YOLOv8	Panoramic	RBL (%), Periodontitis Stage	0.94 (bone level detection, test set)	0.99 (bone level detection, test set)	YOLOv8-based system accurately segmented periodontal landmarks and enabled automated periodontitis staging and grading on panoramic radiographs.
**Karacaoğlu et al., 2023 [43]**	ML-SVM	Periapical	Periodontal defect classification	1.00 (no periodontal defect) 1.00 (periodontal defect is exist)	NA	The proposed AI system can be utilized in the support of clinical practitioners
**Rezallah et al., 2025 [94]**	MobileNetV2 & YOLOv8	Panoramic	PBL	1.00 (MobileNet-V2 recall for PBL detection)	0.88 (MobileNet-V2 binary classification)	Two-stage AI system detected and classified periodontal bone loss severity on panoramic radiographs.
**Tsoromokos et al., 2022 [95]**	Custom CNN (13-layer convolutional neural network)	Periapical	Alveolar bone loss	0.96 (patient-level classification; <33% vs. ≥33% ABL)	0.80 (patient-level classification)	CNN showed moderate to good reliability for detecting and quantifying alveolar bone loss on periapical radiographs.
**Lee et al., 2025 [49]**	Faster-RCNN architecture with RPN, Box Classifier with Inception-ResNet-V2	Bitewing	Alveolar crestal height	NA	0.94	AI application accurately measured alveolar crestal height on bitewing radiographs.
**Cassiano et al., 2025 [96]**	YOLO-v8-pose CNN	Bitewing	RBL	0.739	NA	AI model detected and measured periodontal radiographic bone loss on bitewing radiographs.
**Widyaningrum et al., 2025 [97]**	Mask R-CNN with DenseNet169	Panoramic	RBL, periodontitis staging classification	0.51 (average, external test set)	0.80 (average, external test set)	Hybrid two-stage CNN detected and staged periodontitis on panoramic radiographs with the highest performance in advanced stages.
**Chen et al., 2023 [45]**	YOLOv2& AlexNet-based CNN	Periapical	Classification of peri-implant bone destruction	0.905 (implant detection recall)	0.9045 (peri-implantitis damage classification)	CNN-based system accurately assessed peri-implantitis damage on periapical radiographs.
**Kong et al., 2024 [98]**	SRGAN & U-Net & Canny edge detection	Panoramic	PBL (Segmentation)	0.887 (SRGAN-enhanced images)	0.904 (SRGAN-enhanced images)	SRGAN-enhanced images improved U-Net segmentation accuracy for periodontal bone loss on panoramic radiographs.
**Bilal et al., 2024 [35]**	Custom PDCNET CNN	Panoramic	Periodontal Condition Classification (Periodontal/Non-Periodontal)	0.9839	0.9839	PDCNET accurately classified periodontal disease on dental radiographs.
**Liu et al., 2025 [99]**	GPT-4o	Panoramic	PBL (Horizontal + Vertical Loss)	NA	NA	GPT-4o showed high performance for detecting radiopaque conditions (e.g., dental implants) on panoramic radiographs but poor performance for radiolucent conditions.
**Lee et al., 2025 [46]**	Ensemble-based YOLOv8	Panoramic	Peri-implant defect morphology and defect severity	0.853	0.8533	DL accurately classified peri-implantitis defect morphology and severity on panoramic radiographs.
**Ragab et al., 2025 [100]**	Modified YOLOv7-M	Panoramic	RBL Staging (Healthy/Mild/Moderate/Severe)	0.871	NA	Modified YOLOv7 accurately detected periodontal bone loss on panoramic radiographs with high precision and recall.
**Abu et al., 2025 [37]**	YOLOv8 & MobileNet & EfficientNet & InceptionV3 & XceptionNet & ResNet50	Bitewing	Radiographic intrabony defect angle (>37° vs. <37°).	0.9512	0.9512	DL model accurately classified intrabony defect angles (>37° vs. ≤37°) on bitewing radiographs with high accuracy (~95%).
**Lee et al., 2022 [36]**	U-Net with ResNet-34 encoder & U-Net with CNN blocks	Periapical	RBL, periodontitis staging classification	Stage I: 0.82 Stage II: 0.93 Stage III: 0.80 No bone loss: 0.96	Stage I: 0.91 Stage II: 0.88 Stage III: 0.99 No bone loss: 0.99	DL model accurately measured radiographic alveolar bone level and assigned RBL stages and periodontitis diagnosis on periapical radiographs.

## 4. Discussion

In this systematic review, the effectiveness of fractal analysis and artificial intelligence-based approaches for the assessment of periodontal bone loss using panoramic and intraoral radiographs was comprehensively evaluated. The findings indicate that both approaches provide meaningful contributions to the evaluation of periodontal tissues; however, there is substantial heterogeneity with respect to the applied methods, imaging protocols, analytical parameters, and reference standards. This methodological heterogeneity makes it difficult to directly compare reported diagnostic performance results and prevents clear conclusions about which approach is clinically more reliable. Furthermore, differences in imaging protocols and ROI definitions limit the consistent applicability of the methods across different patient populations. Therefore, current findings regarding the integration of fractal analysis and AI-based approaches into routine clinical practice should be interpreted with caution. In addition to these differences, the methodological quality of the studies is also an important factor affecting the interpretation of the findings. Indeed, the high risk of bias frequently observed in the QUADAS-2 assessment, particularly in patient selection and index test domains, suggests that the reported diagnostic performance metrics may have been reported more optimistically than in some studies. Limitations in patient selection may lead to non-representative samples, which may not adequately reflect the performance of models in real clinical populations. Similarly, high risk in the index test domain may lead to overestimation of reported criterion values due to methodological factors such as lack of blinded assessment or absence of independent external validation. The extremely high accuracy rates reported in some retrospective studies likely reflect optimistic estimates that may not be fully replicable in real-world clinical scenarios where image quality varies and confounding factors exist. Furthermore, although the review was conducted in accordance with the PRISMA guidelines, the general confidence in the current evidence base is considered moderately acceptable due to methodological heterogeneity, the frequent risk of bias, and limited external validation. Therefore, clinical caution should be exercised when interpreting the current findings.

Fractal analysis has long been used in periodontal and implant-related research because of its ability to quantitatively reveal microstructural bone changes that are difficult to detect visually on radiographic images [101,102,103,104]. Although various approaches for calculating fractal dimension have been described in the literature, the box-counting method is the most widely used and accepted technique, as also emphasized by Kato et al. [10]. Notably, all fractal analysis studies included in this review employed the box-counting method, which is methodologically noteworthy. Nevertheless, fractal analysis results did not consistently demonstrate uniform differences across periodontal conditions. Eser and Sarıbas reported no significant differences in fractal dimension between the gingivitis and periodontitis groups [27]. In contrast, Korkmaz et al. reported significantly lower fractal dimension values in individuals with periodontitis compared with healthy controls [25]. Despite these discrepancies, the studies summarized in Table 2 generally suggest a tendency for FD values to decrease with increasing disease severity [23,24,26,29,30].

Examination of the FD values reported in this review shows that values of approximately 1.21–1.66 were observed in periodontally healthy individuals, whereas lower values were reported in groups with periodontal disease, with FD ranging approximately between 1.02 and 1.86. This wide range appears to be influenced by differences in study design and ROI selection, which may lead to divergent reporting trends. For instance, Mirsha et al. reported a mean FD value of 1.21 ± 0.07 in healthy individuals, which decreased to 1.02 ± 0.11 with the progression of disease stage [24]. Similarly, Soltani et al. reported progressive reductions in fractal dimension in mild, moderate, and severe periodontitis groups, with corresponding values of 1.62, 1.52, and 1.32, respectively [26]. Collectively, these findings suggest that fractal dimension may decrease as alveolar bone microarchitecture deteriorates; however, the considerable variability across studies highlights the sensitivity of fractal analysis to methodological factors.

Significant differences were observed among studies in terms of ROI size (ranging from 15 × 15 pixels to 60 × 60 pixels), anatomical location of the ROI (interdental, molar, premolar, condylar, or gonial regions), and imaging modalities (panoramic vs. periapical radiographs). These differences lead to deviations in fractal dimension values due to variations in the analyzed bone microarchitecture and limit the reproducibility of the results. In addition, the inclusion of peri-implant bone loss assessments in some studies further increases heterogeneity within the study populations, thereby limiting the direct comparability of results. As presented in Table 2, fractal dimension values vary not only according to the presence of disease but also substantially depending on the selected anatomical region. The reporting of different FD values across mandibular molar, premolar, interdental, gonial, and condylar regions suggests that ROI localization is a critical variable that directly influences fractal analysis outcomes. Accordingly, there is a clear need for standardized ROI definitions. The variation in ROI size and location is not merely a methodological detail; it directly affects the reproducibility of the fractal dimension calculation. Larger ROIs may average out microstructural details, while smaller ROIs may be too sensitive to local noise, leading to inconsistent FD values even within the same patient population. Therefore, fixed pixel dimensions and homogeneous imaging protocols are essential to improve comparability.

Furthermore, fractal dimension values appear to reflect not only localized periodontal destruction but also systemic and biological factors. Variables such as diabetes, menopausal status, and age have been reported to influence bone microarchitecture, with corresponding effects on fractal dimension values, as demonstrated in several studies [29,30]. Studies evaluating systemic conditions such as diabetes and menopause have shown that FD values may differ even when periodontal status is similar, suggesting that fractal analysis is sensitive not only to periodontal tissue changes but also to systemic bone metabolism. Therefore, future studies should take systemic factors into account when interpreting fractal analysis findings.

Considering fractal analysis and artificial intelligence approaches together provides a more comprehensive framework for periodontal assessment. While fractal analysis generates quantitative and structural features from radiographic images, artificial intelligence models can leverage such features and/or raw image data to achieve high performance in classification, segmentation, and regression-based tasks. In this respect, the two approaches can be regarded as complementary, though they differ fundamentally in maturity and translational potential. Structurally, fractal analysis is a mathematical method that focuses on quantifying bone microarchitecture and quality using smaller, region-specific ROIs. It is computationally less demanding and does not require training phases, but its scalability is limited by the need for precise, often manual, ROI placement, which affects reproducibility. In contrast, AI approaches, particularly deep learning models, offer superior scalability and the potential for full automation, making them more suitable for screening large populations. However, AI models require massive, annotated datasets for training and are often criticized for their ‘black box’ nature, lacking the biological interpretability inherent in fractal dimension values. While FA provides specific insights into trabecular complexity potentially useful for early disease detection, AI excels in the rapid classification and segmentation of established bone loss patterns.

In the current classification system proposed in 2017 by the American Academy of Periodontology (AAP) and the European Federation of Periodontology (EFP), clinical attachment loss (CAL) is accepted as the primary criterion for staging and grading periodontitis; when CAL measurement is not feasible, radiographic bone loss (RBL) is used as a surrogate measure [105]. However, interpretation of radiographs—particularly in the early stages of disease and in anatomically complex regions—is highly dependent on clinician experience. Deep learning-based approaches have the potential to enhance reproducibility and reduce subjective variability in image interpretation [98].

A substantial number of studies included in this review demonstrate that artificial intelligence models can achieve high performance in detecting periodontal bone loss and staging periodontitis [32,48,56,59,66,70,72,84,86,92,100]. Nevertheless, the performance metrics summarized in Table 3 reveal substantial variability across studies: reported sensitivity ranged from 0.23 to 1.00, accuracy from 0.506 to 1.00, specificity from 0.41 to 0.99, and F1 score from 0.15 to 0.99, while AUC values—when reported—varied between 0.75 and 0.99. For example, a study conducted on 2000 panoramic radiographs reported a sensitivity of 1.00 and an accuracy of 0.98 [66], whereas another study including 500 panoramic images reported a sensitivity of 0.9464 and an accuracy of 0.9027 [71]. In contrast, a study reporting sensitivity as low as 0.23 on panoramic images has also been published [50]. This variability is further compounded by the inconsistent reporting of performance metrics across the literature. As noted in Table 3, while some studies report a comprehensive suite of metrics, including AUC and F1 scores, others rely solely on accuracy. The absence of critical metrics like sensitivity and specificity in some studies masks the model’s ability to distinguish between false positives and false negatives, which is crucial in clinical diagnostics. This selective reporting prevents a robust quantitative meta-analysis and hinders a transparent comparison of model efficacy. This underscores the urgent need for standardized evaluation and reporting frameworks.

In a study performing multiclass periodontal staging, accuracy was reported as 0.762 [72], while another study employing an external test set demonstrated that sensitivity could decrease to as low as 0.51 [97]. These findings suggest that model performance may vary considerably depending on task definition, dataset characteristics, and validation strategies, and that generalizability may therefore be limited.

Several studies have directly compared artificial intelligence models with clinicians and reported comparable or superior performance for AI-based approaches [51,57,58,91]. Jiang et al. [55] and Jundaeng et al. [66] demonstrated that AI models were able to detect early-stage periodontal bone loss with higher accuracy than general dentists. This finding highlights the potential of artificial intelligence as a clinical decision-support tool in early diagnosis and screening. However, many studies also report that model performance varies according to defect type and severity; for instance, some models perform better in detecting localized bone loss, while performance declines for total bone loss, vertical defects, and furcation involvement. This trend is consistent with reports suggesting reduced accuracy in advanced and complex bone defects, potentially due to underrepresentation of severe cases in training datasets [50,55].

As summarized in Table 3, the majority of artificial intelligence applications have been developed using panoramic and intraoral radiographs. A more detailed breakdown shows that 41 studies were conducted on panoramic radiographs, 13 on periapical radiographs, 5 on bite-wing radiographs, and 7 on combined periapical and bite-wing images, with a limited number of studies utilizing CBCT data. This distribution may be related to the widespread use of panoramic imaging in routine clinical practice and its broad anatomical coverage. In contrast, studies based on periapical and bite-wing radiographs tend to focus more on quantitative assessment of alveolar bone levels, whereas CBCT-based studies tend to evaluate complex bone defects and furcation involvement.

There is marked heterogeneity in both the performance metrics reported and the reporting practices across the literature. While some studies report only accuracy, others present a broader set of metrics, such as sensitivity, specificity, F1 score, or AUC. Additionally, differences in reference standards—ranging from single-expert annotation to multi-expert or consensus-based evaluations—further complicate cross-study comparisons. This variability underscores the need for standardized evaluation and reporting frameworks in AI-based periodontal research.

Overall, this systematic review demonstrates that both fractal analysis and artificial intelligence-based approaches have the potential to provide objective and reproducible outputs for the assessment of periodontal bone loss. However, methodological diversity and heterogeneity in reporting limit the direct comparability and generalizability of the findings.

### Limitations

This systematic review demonstrates that fractal analysis and artificial intelligence-based approaches offer promising results in the assessment of periodontal bone loss, and it also highlights significant research gaps in the current literature. First, there is substantial heterogeneity among the included studies with respect to imaging modalities, ROI definitions, analysis parameters, and reference standards; this limits the direct comparability and generalizability of the results. In fractal analysis studies, variations in ROI size, anatomical localization, and image quality may significantly influence the derived fractal dimension values. In artificial intelligence studies, the inconsistent reporting of performance metrics (e.g., accuracy, sensitivity, specificity, and AUC) and variability in reference standards (single expert, multiple experts, or consensus) complicate the assessment of the clinical significance of findings and the interpretation of results. This selective or incomplete reporting can lead to reporting bias and make meaningful comparisons between studies difficult, resulting in model performance being perceived as higher than it actually is. Furthermore, many studies are retrospective, single-center, and based on relatively small sample sizes. This selective or incomplete reporting can lead to reporting bias and make meaningful comparisons between studies difficult, resulting in model performance being perceived as higher than it actually is. Independent external validation sets and prospective multicenter studies are quite limited. This situation may lead to inadequate representation of advanced and complex bone defects and prevent reliable evaluation of the models’ performance in real clinical settings. The predominance of panoramic radiographs in the included studies further limits the generalizability of the findings to other imaging modalities. Finally, the number of studies integrating fractal analysis with AI-based approaches is limited, and the lack of standardized analysis protocols hinders effective integration of these two methods.

## 5. Conclusions

This systematic review demonstrates that fractal analysis and AI-based approaches have significant potential for the assessment of periodontal bone loss. While fractal analysis provides quantitative information on bone microarchitecture, AI models enable automated, objective, and reproducible evaluations from radiographic data. However, the literature exhibits substantial heterogeneity in imaging modalities, ROI definitions, analysis protocols, reference standards, and reporting practices, which limits the comparability and generalizability of the findings. In the future, standardized ROI definitions, harmonized datasets with external validation, multicenter study designs, and comprehensive performance reporting will enhance clinical applicability. In particular, consistent reporting of AUC, F1 score, and external validation results will enhance comparability between studies and contribute to clinical decision-making processes. Furthermore, future research focusing on developing hybrid models that combine fractal analysis and artificial intelligence approaches, prospective multi-center validation, and integration into clinical decision support systems will reveal the true clinical value of these methods in periodontal disease management.

## Figures and Tables

**Table 1:** Database-specific search syntax.

**Web of Science**	*TS = (“fractal analysis” OR “fractal dimension” OR fractal OR “texture analysis” OR radiomics OR “artificial intelligence” OR “machine learning” OR “deep learning” OR “computer-aided” OR AI) AND TS=(periodontitis OR “periodontal disease” OR “periodontal bone” OR “alveolar bone loss”) AND TS=(radiograph OR “dental radiograph” OR periapical OR bitewing OR panoramic OR OPG OR CBCT OR “cone beam” OR “intraoral radiograph”)*
**PubMed**	(“fractal analysis” [Title/Abstract] OR “fractal dimension” [Title/Abstract] OR “fractal” [Title/Abstract] OR “fractals” [MeSH Terms] OR “texture analysis” [Title/Abstract] OR “radiomics” [Title/Abstract] OR “artificial intelligence” [Title/Abstract] OR “machine learning” [Title/Abstract] OR “deep learning” [Title/Abstract] OR “computer-aided” [Title/Abstract] OR “AI” [Title/Abstract]) AND (“periodontitis” [Title/Abstract] OR “periodontal disease” [MeSH Terms] OR “periodontitis” [Title/Abstract] OR “alveolar bone loss” [Title/Abstract] OR “periodontal bone” [Title/Abstract]) AND (“radiograph” [Title/Abstract] OR “dental radiography” [MeSH Terms] OR “periapical radiograph” [Title/Abstract] OR “bitewing” [Title/Abstract] OR “panoramic” [Title/Abstract] OR “OPG” [Title/Abstract] OR “CBCT” [Title/Abstract] OR “cone-beam” [Title/Abstract] OR “intraoral radiograph” [Title/Abstract])

## Data Availability

Data are contained within the article or Supplementary Materials.

## Supplementary Materials

The following supporting information can be downloaded at https://www.mdpi.com/article/10.3390/diagnostics16050782/s1, Table S1: Performance metrics (accuracy, sensitivity, precision, specificity, AUC, recall, and F1 score) of the artificial intelligence–based studies included in the review; Table S2: PRISMA 2020 checklist [106]; Figure S1: Risk of bias assessment for included studies using QUADAS-2 domains.
