# Supplementary material for: Fractal Analysis and Artificial Intelligence for Radiographic Detection of Periodontal Bone Loss: A Systematic Review
Source: Diagnostics (Basel). 2026 Mar 5;16(5):782. doi: 10.3390/diagnostics16050782 (PMC12985234; doi:10.3390/diagnostics16050782)
Supplement: Supplementary file 1 [file diagnostics-16-00782-s001.zip › Supplementary Table S1.docx]

| Authors | Al Algorithm | Task | Sample Size (n) | Comparator/Ground Truth | Specifity | Precision | Recall | F1 Score | AUC |
| --- | --- | --- | --- | --- | --- | --- | --- | --- | --- |
| Mardini et al., 2024 [50] | Deep CNN | Hybrid | 500 panaromic radiographs | Three Periodontists | NA | 0.11 | 0.26 | 0.15 | NA |
| Yu et al., 2024 [51] | SegFormer | Hybrid | 775 images | Two Experienced Dentists | NA | 0.7625 | 0.7347 | 0.7458 | NA |
| Kabir et al., 2022 [52] | U-Net with ResNet-34 | Hybrid | 150 periapical radiographs | 2 board-certified periodontists and 1 periodontics resident | Stage I 0.93 Stage II 0.95 Stage III 0.92 | NA | NA | NA | Stage I 0.89 Stage II 0.90 Stage III 0.90 |
| Kearney et al., 2022 [22] | GAN (CNN) Deep Lab V3+DETR | Measurement | 103,914 images | 1 Periodontist, 2 General Dentist | NA | NA | NA | NA | NA |
| Li et al., 2024 [53] | Unet & TransUNet & TA-Net & CE-Net & DS-TransUNet | Hybrid | 6,580 panoramic radiographs | Model-to-Model Comparison | 0.9966 (periodontitis segmentation, TA-Net, Test3) | NA | 0.9736 | 0.9856 (micro-F1, classification, ResNet, mixed dataset) | NA |
| Zhao et al., 2022 [54] | Modified BCDU-Net& Dual-loss autoencoder& DNN | Classification | 1,188 panoramic radiographs | Image-based model (OPG) vs Clinical-data model (EHR) vs Multimodal fusion model (OPG+EHR) | 0.83 (Chapter III); 0.80 (Chapter VI); 0.68 (Chapter IX) | 0.85 (Chapter III); 0.81 (Chapter VI); 0.71 (Chapter IX) | 0.93 (Chapter III); 0.84 (Chapter VI); 0.77 (Chapter IX) | 0.89 (Chapter III); 0.83 (Chapter VI); 0.74 (Chapter IX) | 0.92 (Chapter III); 0.87 (Chapter VI); 0.78 (Chapter IX) |
| Li et al., 2025 [31] | HC-Net, HC-Net+ | Hybrid | 10,400 panoramic radiographs | Two periodontal specialists, two dental surgeons, and two dental students | 0.88 | 0.77 | 0.77 | 0.77 | NA |
| Jiang et al., 2022 [55] | DL-UNET & YOLOv4 | Hybrid | 640 panoramic radiographs | Three Periodontists | 0.98 | 0.90 | 1.00 | 0.90 | NA |
| Jundaeng et al., 2025 [56] | YOLOv8 (CNN) | Measurement | 2,000 panoramic radiographs | Not reported | 60.2–85.2% (tooth presence detection; model-dependent) | 85.6–94.0% (PPV; tooth presence detection) | 85.8–93.1% | NA | NA |
| Camlet et al., 2025 [57] | ChatGPT | Hybrid | 10 panoramic radiographs | Three Clinicians | 0.99 (total alveolar bone loss segmentation) | 0.55 (total alveolar bone loss segmentation) | 0.60 (total alveolar bone loss segmentation) | 0.78 (classification model) | 0.8499 (total alveolar bone loss segmentation) |
| Kurt-Bayrakdar et al., 2025 [38] | nnU-Net v2 | Detection | 502 CBCT volumes | Experienced Periodontist and Two experienced oral and maxillofacial radiologists | NA | NA | NA | NA | NA |
| Butnaru et al., 2025 [58] | CNNs | Hybrid | 6 panoramic radiographs | Large Expert Panel (60 clinicians) | NA | NA | NA | NA | NA |
| Liu et al., 2025 [59] | Mask R-CNN & U-Net | Detection | 238 panoramic radiographs | Not reported | NA | NA | NA | NA | 0.823 (unaided, all radiographs); 0.830 (DL-aided, all radiographs) |
| Alghaihab et al., 2025 [60] | Denti.AI (DL) ResNeT, FPN | Hybrid | 26 intraoral radiographs | Reference standard: a board-certified oral and maxillofacial radiologist and two periodontists Comparator: General dentists (n=10) | NA | NA | NA | 0.889 (Suzhou, Macro F1); 0.812 (Zhongshan, Macro F1) | NA |
| Li et al., 2021 [61] | Deetal-Perio, R-CNN | Classification | 506 panoramic radiographs | Expert dentist | NA | NA | NA | NA | NA |
| Resul et al., 2025 [32] | APD-FFNet | Detection | 337 panoramic radiographs | Single Periodontist | 0.71 | NA | 0.96 | NA | 0.85 |
| Mema et al., 2025 [62] | Diagnocat | Classification | 104 panoramic radiographs; | Three Multidisciplinary Clinicians | NA | 0.98 | 0.98 | 0.98 | 0.98 |
| Shetty et al., 2024 [39] | ResNet50 & ResNet101 & ResNet101V2 | Hybrid | 285 CBCT images | Model-to-Model Comparison | 0.88 ± 0.03 | 0.88 ± 0.03 (PPV) | 0.86 ± 0.03 | 0.87 ± 0.01 | 0.92 ± 0.02 |
| Chang et al., 2022 [63] | DL-multitasking InceptionV3 model | Detection | 1,832 periapical radiographs | Three Periodontists | 0.847 | NA | 0.801 | NA | NA |
| Zadrozny et al., 2022 [64] | Diagnocat AI (CNN-based system) | Classification | 30 panoramic radiographs | Three Independent Evaluators | 0.791 | 0.73 | 0.73 | 0.73 | NA |
| Alotaibi et al., 2022 [65] | DL-CNN-based model VGG-16 | Hybrid | 1,724 periapical radiographs | Periodontist | 0.98 | 0.90 | 1.00 | 0.90 | NA |
| Jundaeng et al. 2025 [66] | YOLOv8 (CNN) | Segmentation | 2,000 panoramic radiographs | One periodontist, one GP, and one periodontitis expert (over 10 years of experience) | NA | 0.76 | 0.75 | 0.76 | NA |
| Saylan et al., 2023 [67] | YOLOv5x | Detection | 685 panoramic radiographs | Two-Specialist (radiologist + periodontist) | 93.1% (marginal bone loss) | 94.9% (PPV, marginal bone loss) | 91.1% (marginal bone loss) | NA | NA |
| Ibraheem et al., 2025 [68] | Second Opinion | Hybrid | 300 intraoral periapical radiographs | Two trained oral radiologists | NA | 0.694 | 0.611 | NA | NA |
| Vollmer et al., 2023 [69] | Mask R-CNN & ResNet-50-FPN | Detection | 1,414 panoramic radiographs | Two Dentists | NA | 0.86 | 0.84 | 0.85 | 0.88 |
| Ryu et al., 2024 [70] | Faster R-CNN | Detection | 4,083 panoramic radiographs | Two Dentists | 0.8094 | 0.9138 | 0.9464 | 0.9298 | NA |
| Putra et al., 2025 [71] | YOLOv8 | Hybrid | 500 panoramic radiographs | a radiologist and a periodontist | NA | NA | NA | NA | NA |
| Kong et al., 2023 [72] | Two-stage PDCNN architecture | Hybrid | 1,747 panoramic radiographs | Model-to-Model Comparison | 0.86 (periapical); 0.90 (bitewing) | 0.83 (PPV, periapical); 0.88 (PPV, bitewing) | 0.76 (periapical); 0.65 (bitewing) | NA | NA |
| Alghaihab et al., 2025 [73] | DL-ResNet CNN | Hybrid | 39 intraoral radiographs | Oral and maxillofacial radiologist and 3 periodontist | NA | NA | NA | NA | NA |
| Chen et al., 2023 [74] | CNN: YOLOv5 including VGG-16 and U-Net | Segmentation | 8,000 periapical radiographs | 5 senior clinical dentists with periodontal training and radiology backgrounds | NA | NA | NA | NA | NA |
| Su et al., 2024 [47] | Res-Net | Classification | 389 CBCT | A physician, an imaging technician, an associate chief physician | 0.699–0.745 | 0.876–0.891 (PPV) | 0.898–0.913 | NA | 0.899–0.918 |
| Dujic et al., 2023 [75] | Vit Base&VitLarge&BEiTBase&BEiT Large&DeiTbase | Classification | 21,819 periapical radiographs | Dentist | 0.9473 | 0.9337 | 0.9441 | 0.9333 | NA |
| Vilkomir et al., 2024 [40] | ResNet-18 | Classification | 1,078 periapical radiographs | Not reported | 0.98 | 0.97 | 0.95 | NA | 0.99 |
| Yavuz et al., 2024 [33] | YOLOv8-cls (Ultralytics) | Classification | 1120 periapical radiographs  1498 bitewing radiographs | Three periodontologist + one radiologist | 0.7162 (bitewing); 0.7500 (periapical) | 0.7439 (bitewing); 0.7500 (periapical) | 0.8243 (bitewing); 0.7500 (periapical) | 0.7821 (bitewing); 0.7500 (periapical) | NA |
| Piroonsan et al., 2025 [41] | InceptionV3, InceptionResNetV2, ResNet50V2, MobileNetV3Large, EfficientNetV2B1, and VGG19 | Hybrid | 1,369 intraoral radiographs | Model-to-Model Comparison | 0.88 (VGG19, dataset B3) | 0.82 (VGG19, dataset B3) | 0.67 (VGG19, dataset B3) | 0.74 (VGG19, dataset B3) | 0.77 (MobileNetV3Large, dataset B3); 0.75 (VGG19, dataset B3) |
| Ezhov et al., 2021 [76] | CNN | Detection | 1346 CBCT | Dentists | 0.9899 | NA | 0.9239 | NA | NA |
| Schulze et al., 2024 [42] | CNN-Diagnocat | Classification | 38 CBCT | Three general dentists | NA | NA | NA | NA | NA |
| Ayyildiz et al., 2024 [77] | ResNet50& DenseNet121& InceptionV3 | Detection | 2,533 panoramic radiographs | Periodontology specialty student and a periodontist | 0.944 | 0.880 | 0.833 | 0.856 | 0.888 |
| Bahadır et al., 2024 [78] | DentisToday | Segmentation | 50 panoramic radiographs; | Undergraduate students, final-year dentistry students | NA | NA | NA | NA | NA |
| Widyaningrum et al., 2022 [79] | Multi-Label U-Net & Mask R-CNN | Hybrid | 100 panoramic radiographs | Two Experts (Dentist + Periodontist) | NA | 0.856 (Mask R-CNN, average) | 0.88 | 0.866 (Mask R-CNN, average) | NA |
| Dai et al., 2024 [80] | AlexNet, VGG16, ResNet18 with multiple classification algorithms (RF, SVM, NB, LR, KNN) | Hybrid | 11,120 periapical radiographs | Three blinded periodontists | 0.779 (PER-AlexNet model) | NA | 0.915 | NA | 0.936 (PER-AlexNet model) |
| Chen et al., 2024 [81] | U-Net + Mask-RCNN (deep CNN pipeline) | Classification | 336 periapical radiographs | Three calibrated board-certified periodontists | 0.638 (cut-off 0.15); 0.896 (cut-off 0.33) | 0.742 (PPV, cut-off 0.15); 0.638 (PPV, cut-off 0.33) | 0.97 (cut-off 0.15); 0.952 (cut-off 0.33) | 0.841 (cut-off 0.15); 0.764 (cut-off 0.33) | 0.946 (cut-off 0.15); 0.968 (cut-off 0.33) |
| Erturk et al., 2025 [82] | YOLOv8 | Classification | 1,752 bite-wing radiographs | Dentist (2 years of experience in oral radiology) | NA | 0.81742 (test, average) | 0.80883 (test, average) | 0.81090 (test, average) | NA |
| Zhang et al., 2023 [44] | CNN (ResNet-50) | Classification | 529 periapical radiographs 551 panoramic radiographs | Two Implant Surgeons | NA | 0.85 (hybrid model) | 0.88 (hybrid model) | 0.85 (hybrid model) | 0.972 (failure with marginal bone loss); 0.947 (failure without marginal bone loss); 0.975 (success) |
| Mao et al., 2023 [83] | CNN (GoogLeNet; AlexNet; VGG19; Inception v3) | Hybrid | 300 periapical radiographs | Model-to-Model Comparison | NA | 0.916 (GoogLeNet) | 0.956 (GoogLeNet) | 0.935 (GoogLeNet) | NA |
| Li et al., 2025 [84] | YOLOv8 | Classification | 558 panoramic radiographs | Three Periodontists | NA | 0.76 (overall) | 0.64 (overall) | 0.68 (overall) | 0.79 (micro-average) |
| Liu et al., 2023 [85] | CNN (PAR-CNN model based on AlexNet) | Hybrid | 2,275 panoramic radiographs | Three periodontal experts, Three general dental practitioners | 0.784 | NA | 0.795 | NA | 0.843 *(primary test set); 0.793 (external test set) |
| Xue et al., 2024 [86] | YOLOv8 & Mask R-CNN & TransUNet | Hybrid | 320 panoramic radiographs | Three Experienced Dentists | NA | NA | NA | NA | NA |
| Shon et al., 2022 [48] | U-Net & YOLOv5 | Measurement | 87 panoramic radiographs | Two Specialist | NA | 0.732 (mean) | 0.805 (mean) | 0.696 (mean) | NA |
| Almarghlani et al., 2025 [87] | ML, Second Opinion® | Classification | 10 intraoral radiographs | General Practitioners , periodontist specialists, prosthodontics specialists and other dental specialties. | NA | NA | NA | NA | NA |
| Hoss et al., 2023 [88] | ResNet-18, MobileNet V2, ConvNeXT/large | Segmentation | 21,819 periapical radiographs | 7 dentists | 0.712 (highest: ConvNeXT/base) | 0.880 (PPV, ConvNeXT/base) | 0.907 | NA | 0.913 (ConvNeXT/large) |
| Kurt-Bayrakdar et al., 2024 [89] | CNN U-Net | Hybrid | 1121 panoramic radiographs | Multidisciplinary Expert Panel (4 experts) | NA | 0.995 (total bone loss) | 1.000 (total bone loss) | 0.997 (total bone loss) | 0.951 (total bone loss) |
| Li et al., 2024 [34] | YOLOv4 + CNN (AlexNet) | Hybrid | 210 bitewing radiographs | AlexNet, ResNet101, ResNet50, EfficientNetV2B0 (Model-to-model comparison) | NA | NA | NA | NA | NA |
| Ertas et al., 2023 [90] | kNN, ANN, SVM, RF, NB, LR (clinical data); DenseNet121, EfficientNetB0, InceptionV3, ResNet50, VGG16 (image DL); hybrid CNN+ML | Hybrid | 144 panoramic radiographs | Model-to-Model Comparison | NA | NA | NA | NA | 0.975 (random forest, clinical data – staging) |
| Orhan et al., 2023 [91] | Diagnocat CNN Software | Hybrid | 100 panoramic radiographs | Three OMF Radiologists | 0.935 | 0.772 | 0.818 | NA | NA |
| Amasya et al., 2023 [92] | Mask R-CNN (ResNet-101 backbone) + Cascade R-CNN | Hybrid | 6,000 panoramic radiographs | Three Clinicians | NA | 0.971 (binary bone loss detection, overall) | 0.999 | 0.985 (binary bone loss detection, overall) | NA |
| Do et al., 2025 [93] | YOLOv8 | Classification | 500 panoramic radiographs | A radiologist | 0.99 (bone level detection, test set) | 0.95 (bone level detection, test set) | 0.94 (bone level detection, test set) | 0.95 (bone level detection, test set) | NA |
| Karacaoğlu et al., 2023 [43] | ML-SVM | Hybrid | 87 periapical radiographs | A periodontist | NA | NA | NA | NA | NA |
| Rezallah et al., 2025 [94] | MobileNetV2 & YOLOv8 | Hybrid | 817 panoramic radiographs | Expert OMF Radiologists | NA | 0.74 (YOLOv8) | 0.70 (YOLOv8) | NA | NA |
| Tsoromokos et al., 2022 [95] | Custom CNN (13-layer convolutional neural network) | Hybrid | 446 periapical radiographs | Not Reported | 0.41 (patient-level classification) | NA | NA | NA | NA |
| Lee et al., 2025 [49] | Faster-RCNN architecture with RPN, Box Classifier with Inception-ResNet-V2 | Hybrid | 550 bitewing radiographs | 56 different dental professionals/an oral radiologist | NA | NA | NA | NA | NA |
| Cassiano et al., 2025 [96] | YOLO-v8-pose CNN | Hybrid | 595 bitewing radiographs | General Dentist | NA | 0.611 | 0.739 | 0.6689 | NA |
| Widyaningrum et al. 2025 [97] | Mask R-CNN with DenseNet169 | Hybrid | 600 panoramic radiographs | A dentist, a dental student based on consensus with an oral radiologist | 0.88 (average, external test set) | 0.59 (average, external test set) | 0.51 | 0.53 (average, external test set) | NA |
| Chen et al., 2023 [45] | YOLOv2& AlexNet-based CNN | Segmentation | 456 periapical radiographs | Three experienced dentists | 0.78 (true negative rate for YOLOv2) | 0.95 (implant detection precision) | 0.905 | NA | NA |
| Kong et al., 2024 [98] | SRGAN & U-Net & Canny edge detection | Classification | 698 panoramic radiographs | Model-to-Model Comparison | 0.920 (SRGAN-enhanced images) | NA | NA | 0.91 (Dice similarity coefficient) | NA |
| Bilal et al., 2024 [35] | Custom PDCNET CNN | Vision-Language Classification | 1,026 dental radiographs | Model-to-Model Comparison | NA | 0.9839 | 0.9839 | 0.9831 | 0.9879 |
| Liu et al., 2025 [99] | GPT-4o | Hybrid | 50 panoramic radiographs | Two OMF Radiologists | NA | NA | NA | NA | NA |
| Lee et al., 2025 [46] | Ensemble-based YOLOv8 | Hybrid | 1,075 panoramic radiographs | Two periodontal surgeon | NA | 0.855 | 0.853 | 0.854 | NA |
| Ragab et al., 2025 [100] | Modified YOLOv7-M | Hybrid | 1,747 panoramic radiographs | Not reported | NA | 0.917 | 0.871 | 0.925 | NA |
| Abu et al., 2025 [37] | YOLOv8& MobileNet& EfficientNet& InceptionV3& XceptionNet& ResNet50 | Hybrid | 148 bitewing radiographs | Not reported | NA | 0.9525 | 0.9512 | 0.9518 | NA |
| Lee et al., 2022 [36] | U-Net with ResNet-34 encoder& U-Net with CNN blocks | Classification | 693 periapical radiographs | Three independent examiners | Stage I: 0.97 Stage II: 0.86 Stage III: 0.99 No bone loss: 1.00 | NA | NA | NA | Stage I: 0.89 Stage II: 0.90 Stage III: 0.90 No bone loss: 0.98 |
